# Pathogenicity and vaccine efficacy of two virulent infectious laryngotracheitis virus strains in Egypt

**DOI:** 10.1186/s12917-022-03458-3

**Published:** 2022-09-26

**Authors:** Mohamed El-Saied, Magdy M. El-Mahdy, Mahmoud Bayoumi, Reem A. Soliman, Marwa. F. Elsayed, Ezz El-Din Sakr, Mostafa Bastami, Munir M. El-Safty, Mohamed Shaalan

**Affiliations:** 1 Department of Pathology, Faculty of Veterinary Medicine, Cairo University, Giza, 12211 Egypt; 2 Department of Virology, Faculty of Veterinary Medicine, Cairo University, Giza, 12211 Egypt; 3 Central Laboratory for Evaluation of Veterinary Biologics, Abbasia, Cairo, 11381 Egypt; 4 Department of Poultry Diseases, Faculty of Veterinary Medicine, Cairo University, Giza, 12211 Egypt

**Keywords:** ILTV, Histopathology, Recombinant strain, CEO-like strain, gC gene, Egypt

## Abstract

Infectious laryngotracheitis (ILT) is an economically crucial respiratory disease of poultry that affects the industry worldwide. Vaccination is the principal tool in the control of the disease outbreak. In an earlier study, we comprehensively characterized the circulating strains in Egypt and identified both CEO-like and recombinant strains are dominant. Herein, we investigated the pathogenicity of two virulent strains representing the CEO-like (Sharkia_2018) and recombinant strain (Qalubia_2018). Additionally, we evaluated the efficacy of different commercial vaccines (HVT-LT, CEO, and TCO) against the two isolates in terms of the histopathological lesion scores and the viral (gC) gene load. A total of 270 White Leghorn-specific pathogen-free male chicks were divided into nine groups of 30 birds, each housed in separate isolators. Birds were distributed as follows; one group was non-vaccinated, non-challenged, and served as a negative control. Two groups were non-vaccinated and infected with the two isolates of interest and served as a positive control to test the pathogenicity. Six groups were vaccinated and challenged; two groups were vaccinated with vector vaccine at one day old. The other four groups were vaccinated with either the CEO- or TCO- vaccine (two groups each) at four weeks of age. Three weeks after vaccination, birds were infected with the virulent ILTV isolates. The larynx, trachea, and harderian gland samples were taken at 1, 3, and 7 days post-infection for histopathological lesion score and molecular detection. Notably, The recombinant strain was more virulent and pathogenic than CEO-like ILTV strains. Moreover, the TCO vaccine was less immunogenic than the vector and CEO vaccines.

**Supplementary Information:**

The online version contains supplementary material available at 10.1186/s12917-022-03458-3.

## Introduction

Infectious laryngotracheitis (ILT) is a worldwide respiratory disease that causes severe economic losses in the poultry industry. ILT causes high mortality, decreased egg production, and predisposition to other respiratory pathogens [1–3]. The disease is caused by gallid herpesvirus I (GaHV-1), a member of the genus *Iltovirus*, subfamily *Alphaherpesvirinae* which belongs to the family *Herpesviridae* [4].

Two categories of available vaccines are used for protection against the disease: live attenuated and recombinant viral vector vaccines. The first generation of live GaHV-1 vaccines was present in the 1960s. The live vaccines were attenuated by several passages in either the embryonated eggs known as chicken embryo origin (CEO) [5] or attenuated by several passages in tissue culture known as tissue culture origin (TCO) [6]. Even though the live vaccines have been shown to provide strong protection [7, 8], this vaccine category can easily revert to virulence leading to vaccine outbreaks [9]. Furthermore, the reverted strains showed increased virulence after bird-to-bird transmission. Notably, the CEO vaccines demonstrated more respiratory lesions and mortality than TCO vaccines, which confirm different levels of attenuation between live vaccines. Moreover, molecular assays revealed that both the CEO and TCO vaccines were transmitted horizontally to non-vaccinated birds. Additionally, the CEO vaccines were faster in replication and transmission compared with the TCO vaccines [10].

Epizootics of the infection have recently emerged in the United States (USA), Europe, Australia, and Egypt, which were found to be derived from the CEO vaccines that regained virulence and became the primary source of outbreaks [3, 11–14]

In addition to the live viral vaccines, viral vector vaccines (also known as recombinant vaccines) were generated to fend off ILT. Two commercial viral vector vaccines are available, which differ according to the vector backbone. The immunogenic proteins are inserted on the fowlpox virus (FPV) or herpesvirus of turkeys (HVT) vector. The FPV viral vector vaccine carries glycoprotein B and UL32 genes of GaHV-1 (FPV-LT) [15]. In comparison, HVT viral vector vaccine carries glycoprotein D and I, which form part of the outer coat of GaHV-1 that provokes immunity against both GaHV-1 and Marek's disease (HVT-LT) [12, 16]. Both types of viral vector vaccines are administered on either 18-day-old chicken embryos through *in ovo* route or one-day-old chicks through the subcutaneous route. However, the FPV vector vaccine can also be administered with a wing web vaccination [12]. The lack of bird-to-bird horizontal transmission abolishes the revert to virulence, which has an essential advantage of FPV-LT and HVT-LT. This vaccination category improves birds' performance with diminished clinical signs against ILT compared with CEO- and TCO-live vaccines [15–17]. Various experimental studies proved that this vaccine category could not reduce virus shedding as live viral vaccines [18, 19].

In the USA, the CEO vaccines successfully prevented large-scale disease outbreaks in broiler farms [12, 20]. However, several outbreaks were ignited in different countries, including Australia and Egypt. These outbreaks were caused by the exchange of genetic material resulting in recombination between vaccinal strains with high transmission rates. Virulent recombinant strains of GaHV-1 were isolated and recorded in many Australian outbreaks [21].

Vis-à-vis in Egypt, Shehata et al. [11] investigated the outbreaks between 2007 and 2010. They characterized ILTV strains and found them related to the CEO vaccine with increased virulence through bird-to-bird transmission. Our group comprehensively characterized ILT outbreaks in 30 farms in 8 governorates in Egypt between 2018 and 2019. Through molecular assays, we genotyped all detected strains using sequencing of three envelope glycoprotein genes: gD, gG, and gJ, and the ICP4 gene [14]. Five prototype strains with unique sequences were noticed out of all characterized strains. These five strains were further genotyped into two groups according to phylogenetic analysis of ICP4 and gJ into recombinant ILTV strains (3 prototype strains) and CEO vaccine-like ILTV strains (3 prototype strains) [3, 14]. Interestingly, the outbreaks caused by the recombinant strains were more severe than the CEO vaccine-like strains.

Herein, we aim to comprehensively investigate the pathogenicity of two virulent circulating strains responsible for outbreaks in Egypt, one representative of recombinant strains and one representing CEO-like strains. Furthermore, we aim to evaluate different commercially available and licensed vaccines against these two isolates in terms of the clinical signs, histopathological lesion scores, and the viral genome load.

## Materials and methods

### ILTV vaccines and isolates

Three vaccines were used in this study {CEO vaccine Trachivax (Intervet/Schering Plough Animal Health), TCO vaccine LT-IVAX (Intervet/Schering PloughAnimal Health), and vector vaccine (Viral vector HVT-LT vaccine Innovax ILT, Intervet/Schering-Plough Animal Health)}. The vaccines were prepared and administered as recommended by their respective manufacturers. Further to our recent molecular characterization assays, out of the five prototype strains identified in the latest outbreaks in Egypt, two strains only were selected for further pathological characterization and infection experiments (Qalubia_2018 as representative of the recombinant strain and Sharika_2018 as a representative of the CEO-like strain) [14]. Both strains were adapted to grow successively on chicken embryo fibroblast (CEF) cells prepared from 10-day-old SPF chicken embryos (KoamOsheim, El-Fayoum province, Egypt). Both strains were isolated and titrated, and the titers were expressed as the 50% tissue culture infectious dose (TCID_50_) based on the cytopathic effect (CPE) and estimated using Reed and Muench titration method [22].

### Experimental design

A total of 270 White Leghorn-specific pathogen-free chicks were divided into nine groups of 30 birds each (KoamOsheim, El-Fayoum province, Egypt) and were housed in separate isolators. The chicks were fed with appropriate ration and water ad libitum. This study was conducted according to ARRIVE 2.0 guidelines and approved by the Institutional Animal Care and Use Committee (IACUC) in the Faculty of Veterinary Medicine, Cairo University (VetCU01102020217). Birds were divided into three main categories: (i) non-vaccinated non-infected group (*n* = 30). This group was kept as a negative control in a separate unit without any treatment throughout the entire experiment. (ii) non-vaccinated infected group (*n* = 60) (further divided into two equal subgroups; received either recombinant isolate (RS) or CEO-like vaccine isolate (CS)) (iii) vaccinated infected group (*n* = 180; six subgroups, each containing 30 birds) as indicated in Table 1. At one day of age, two sub-groups were inoculated subcutaneously in the neck region with 0.2 ml Innovax-ILT (vector vaccine). At four weeks of age, the other four groups were inoculated with 0.05 ml in the open eye with either CEO vaccine (two sub-groups) or TCO vaccine (two sub-groups). The designated vaccines were administered with the manufacturer's specified dose, Trachivax, and LT-IVAX, respectively. Three weeks after vaccination, the non-vaccinated infected group (i.e. group (ii)) and the vaccinated-infected group (i.e. group (iii)) were inoculated intraocularly with 10^4^ TCID_50_ /ml with either the recombinant or CEO-like isolate (in a total volume of 0.1 ml).

**Table 1:** Experimental groups and vaccine types utilized in this study

Groups (30 bird per group)	Vaccine	Challenge at 51 days old (0.1 ml Intraocular)	Note
Group 1(N)	Not vaccinated	Not challenged	Control negative
Group 2(CS)	Not vaccinated	10^4^ TCID_50_/ml	Challenged with CEO like strain
Group 3(RS)	Not vaccinated	10^4^ TCID_50_/ml	Challenged with Recombinant strain
Group 4 (VEC + CS)	Innovax-ILT^a^	10^4^ TCID_50_/ml CEO like strain	Vaccinated and challenged
Group 5(VEC + RS)	Innovax-ILT^a^	10^4^ TCID_50_/ml recombinant strain	Vaccinated and challenged
Group 6(CEO + CS)	CEO vaccine^b^ (Trachivax)	10^4^ TCID_50_/ml CEO like strain	Vaccinated and challenged
Group 7(CEO + RS)	CEO Vaccine^b^ (Trachivax)	10^4^ TCID_50_/ml recombinant strain	Vaccinated and challenged
Group 8(TCO + CS)	TCO Vaccine^b^ (LT-IVAX)	10^4^ TCID_50_/ml CEO like strain	Vaccinated and challenged
Group 9(TCO + RS)	TCO Vaccine^b^ (LT-IVAX)	10^4^ TCID_50_/ml recombinant strain	Vaccinated and challenged

^a^0.2 ml injected S/C at one day old

^b^0.05 ml intraocular at 4 weeks old

### Intratracheal pathogenicity index (ITPI)

The pathogenicity was assessed using the ITPI as determined previously [11]. Briefly, clinical symptoms and mortalities were rated daily for seven days post-infection as follows: (0 = normal, 1 = mild respiratory signs as coughing, sneezing, 2 = moderate respiratory signs as mouth breathing and dyspnea, 3 = death). Indices were evaluated by dividing the totality of the scores over the total number of chickens.

### Sampling

Samples were taken at 1-, 3-, and 7-days post-infection. Three randomly selected birds from each group were examined for clinical signs. Then the birds were euthanized and subjected to histopathological and virological examinations. The larynx, trachea, and the harderian gland were collected and divided, one presented in 10% neutral buffered formalin and the other frozen for histopathological and virological examinations, respectively.

### Histopathological scoring

Tracheal and harderian gland lesions were scored according to Hussein et al. [23], with modification as indicated in Tables 2 and 3, respectively. A Lesion scored 1, indicating mild changes, while scores 2 and 3 were considered moderate changes. Lesions scored 4 to 6 were considered severe changes. Ten random optical fields were examined and scored, and the mean of the ten fields was calculated. The mean for two tissues ± standard error (SE) was determined.

**Table 2:** Criteria for tracheal lesion score

Lesion score	Histopathological changes
Score 0	normal histological structure
Score 1	hyperemia and inflammatory cells infiltration
Score 2	hyperemia, inflammatory cells infiltration, and edema
Score 3	hyperemia, inflammatory cells infiltration, edema, and deciliation with catarrhal exudate
Score 4	slight hyperplasia and deciliation with syncytial cell formation
Score 5	hemorrhagic patches, desquamation, and hyperplasia
Score 6	squamous metaplasia with collagen bundles and mononuclear cell infiltration

**Table 3:** Criteria for harderian gland lesion score

Lesion score		Histopathological changes
Score 0	normal structure of harderian gland	
Score 1	congestion with slight depletion of lymphoid aggregates	
Score 2	moderate depletion of lymphoid aggregates	
Score 3	excessive depletion of lymphoid aggregates	
Score 4	lymphocytolysis accompanied by necrosis, desquamation, and exudation	

### Statistical analysis

Statistical analyses were performed using a one-way factorial analysis of variance (ANOVA). Statistical significance was defined as (*P* ≤ 0.05) using Graph Pad Prism 8.0.

#### Viral genome quantitation

Three days post-infection, viral genome load in the trachea was quantified using a real-time qPCR assay in a duplex assay, which targets the viral gene UL44 (gC) and normalized to host DNA represented by chicken α2-collagen gene as previously determined [19, 20, 24]. Briefly, the relative amount of the viral DNA was calculated as the log10 of 2^− ΔΔ^Ct: ΔΔCt_t_- ΔCt_bt_. Where ΔCt_t_ is the amount of ILTV gene normalized against the amount of the collagen gene from the non-vaccinated-infected and vaccinated-challenged groups of birds. ΔCt_bt_ is the amount of the targeted ILTV gene normalized against the amount of the collagen gene detected before infection [24]. Primer sequences are listed in Table 4. Trachea swabs of three birds from each group were taken. DNA extraction was performed using TRIzol reagent (ThermoFisher, USA). The purified DNA was quantified using NanoDrop™ spectrophotometer (Thermo Scientific™, USA.), A total of 100 ng DNA was utilized for the quantification experiments. The internal chicken control was used to estimate the relative change with comparative Ct values of the target viral gene as determined previously [24]. The detection and amplification were performed using the Applied Biosystems 7500 Fast Real-Time PCR System. The reactions were set up in a final volume of 25 μl as follows: 20 µl of HERA SYBR® Green Master Mix (Willowfort, UK), 1 µl of primer mix (400 nM each), and 4 μl of DNA template. The thermal cycling profile was as follows: 95 °C for 10 min; 40 cycles of 94 °C for 15 s; and 60 °C for 45 s. The reaction was followed by melting curve analysis using 0.3 °C increments starting from 60 °C. Data was collected and analyzed with StepOne™ software–v2.3 (Applied Biosystems, USA).

**Table 4:** Primers of ILTV and α2-collagen gene used in the study

Primers	Sequence (5'- 3')
UL 44-F	CCTTGCGTTTGAATTTTTCTG
UL 44-R	TTCGTGGGTTAGAGGTCTGT
α2-collagen gene -F	GGGAACTGGAGAACCCAATTTT
α2-collagen gene -R	CGTGCCGCTGTCTCTACCAT

## Results

### Intratracheal pathogenicity index (ITPI)

Intra tracheal pathogenicity index was recorded in experimental groups to evaluate the degree of clinical signs in coordination with morbidity and mortality rate. The negative control group showed no mortality and clinical symptoms with zero ITPI as predicted. However, the infected groups with CEO-like strain (CS) and Recombinant strain (RS) exhibited severe clinical signs, including dyspnea and mouth breathing. Morbidity and mortality rates were increased in the infected groups, notably higher in the RS group. The ITPI in the RS group was recorded (1.483) compared with the CS group (1.26) (Table 5). However, the morbidity and mortality rates were reduced in vaccinated infected groups with varying degrees of ITPI, as indicated in (Table 5). Interestingly, the lowest ITPI was recorded in the VEC + CS group in contrast to TCO + RS, which had a high ITPI among vaccinated and infected groups.

**Table 5:** Mortality and morbidity percentages with intratracheal pathogenicity index (ITPI) in the experimental groups

Groups	Mortality %	Morbidity %	ITPI^a^
Negative control	0	0	0.0
CS	10%	75%	1.266667
RS	16.6%	85%	1.483333
VEC + CS	1.6%	26.6%	0.53
VEC + RS	3.3%	30%	0.58
CEO + CS	4.5%	35%	0.633333
CEO + RS	6.6%	48.3%	0.9
TCO + CS	7.3%	45%	0.783333
TCO + RS	8.1%	52%	1

^a^The pathogenicity was assessed using intratracheal pathogenicity index (ITPI). Clinical symptoms and mortalities were rated daily for seven days post-infection as follows: (0 = normal, 1 = mild respiratory signs as coughing, sneezing, 2 = moderate respiratory signs as mouth breathing and dyspnea, 3 = death). Indices were evaluated by dividing the totality of the scores over the total number of chickens. CS: CEO like strain, RS: Recombinant strain, VEC + CS: Vector vaccinated group challenged with CEO like strain, VEC + RS: Vector vaccinated group challenged with recombinant strain, CEO + CS: CEO vaccinated group challenged with CEO like strain, CEO + RS: CEO vaccinated group challenged with recombinant strain, TCO + CS: TCO vaccinated group challenged with CEO like strain, TCO + RS: TCO vaccinated group challenged with recombinant strain

### Histopathological findings in the trachea of the ILTV infected groups

**Fig. 1:**
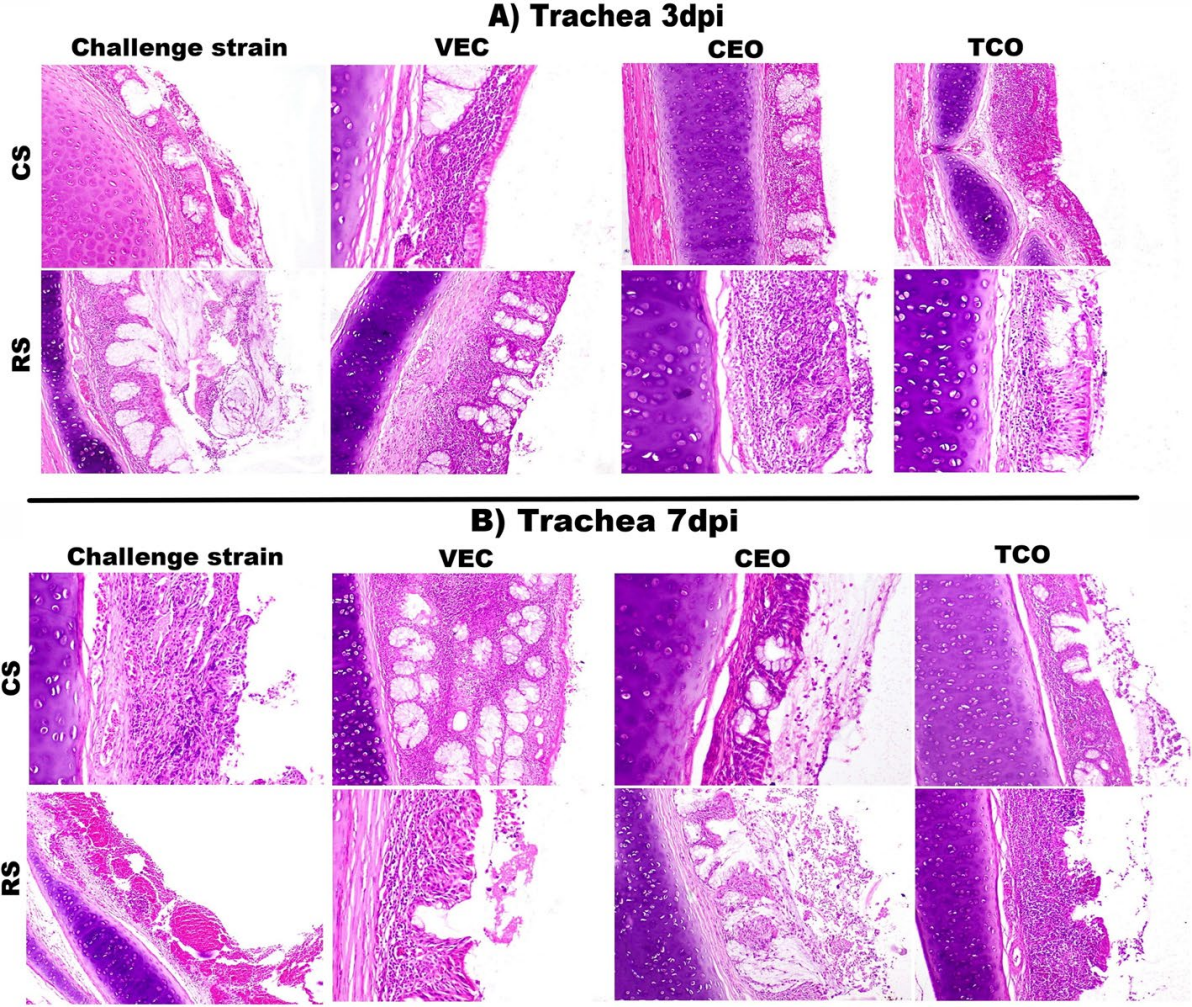
Photomicrographs of H&E-stained tracheal sections of the experimental groups: **A** at 3dpi, CS infected group showing desquamated and inflammatory cell hanged in fibrinous exudate with syncytial cells in the tracheal lumen (H&E,100 X). RS infected group showing more fibrinous exudate in tracheal lumen admixed with syncytial cells and inflammatory cells infiltrate the tracheal mucosa along with congestion (H&E,100 X). In the VEC + CS group, mild inflammatory reaction (H&E,100 X). In VEC + RS showing deciliation with hyperplasia of glands and thickening of tracheal mucosa (H&E,100 X), CEO + CS showed mild to a moderate inflammatory reaction (H&E,100 X), while in the CEO + RS group more infiltration of inflammatory cells with edema, hyperemia, and glandular necrosis (H&E,200 X). TCO + CS shows hyperemic mucosa with inflammatory cells infiltration in its mucosa (H&E,100 X). TCO + RS shows deciliation, desquamation of tracheal lining epithelium with hyperemic mucosa, and infiltrating inflammatory cells (H&E,200 X). **B** at 7dpi, CS group showing severe ulceration of tracheal lining epithelium with mononuclear cells infiltration with hyperemic tracheal mucosa (H&E,200 X). RS shows a diffuse hemorrhagic cast in the tracheal lumen with the complete destruction of tracheal mucosa (H&E,100 X). VEC + CS shows hyperplasia of the mucus gland and hyperactivity with dense inflammatory cells between them (H&E,100 X). VEC + RS shows deciliation with an area of ulceration and hyperemic mucosa (H&E,200 X). CEO + CS showed mild inflammatory reaction with catarrhal exudates, with the presence of heterophils and inflammatory cells in the tracheal lumen (H&E,200 X). CEO + RS presence of a large amount of fibrinoheterophilic exudates in the tracheal lumen with syncytial cell and desquamated epithelium (H&E,100 X). TCO + CS shows a moderate inflammatory reaction of tracheal mucosa also catarrhal exudates and inflammatory cells in their lumen (H&E,100 X). TCO + RS shows severe epithelium necrosis and ulceration with thickening of tracheal mucosa and mononuclear inflammatory cells (H&E,100 X). CS: CEO like strain, RS: Recombinant strain, VEC + CS: Vector vaccinated group challenged with CEO like strain, VEC + RS: Vector vaccinated group challenged with recombinant strain, CEO + CS: CEO vaccinated group challenged with CEO like strain, CEO + RS: CEO vaccinated group challenged with recombinant strain, TCO + CS: TCO vaccinated group challenged with CEO like strain, TCO + RS: TCO vaccinated group challenged with recombinant strain

**Fig. 2:**
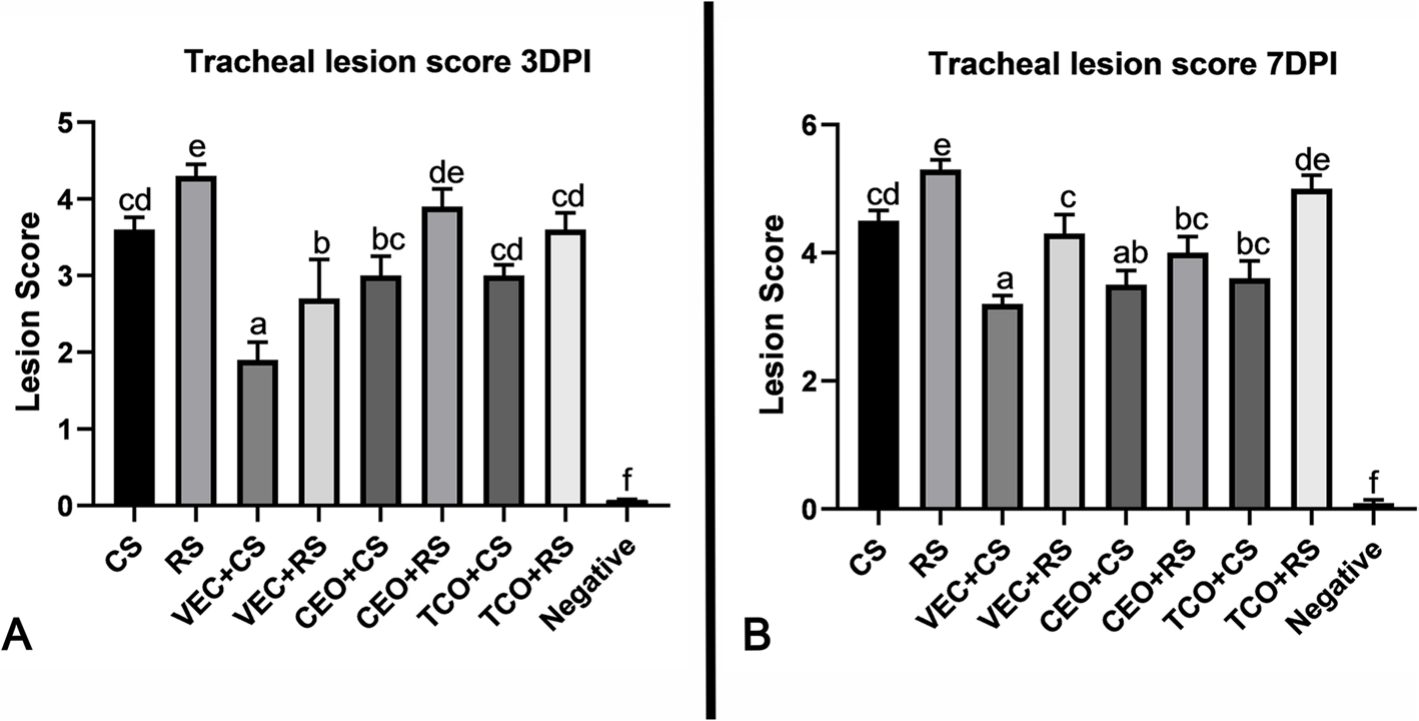
Chart showing tracheal lesion score of the experimental groups: **A** at 3 dpi, **B** at 7dpi, Values expressed as means ± SE (error bars), Statistical analyses were performed using a one-way factorial analysis of variance (ANOVA). Means followed by different letters differ significantly when (*P* ≤ 0.05).CS: CEO like strain, RS: Recombinant strain, VEC + CS: Vector vaccinated group challenged with CEO like strain, VEC + RS: Vector vaccinated group challenged with recombinant strain, CEO + CS: CEO vaccinated group challenged with CEO like strain, CEO + RS: CEO vaccinated group challenged with recombinant strain, TCO + CS: TCO vaccinated group challenged with CEO like strain, TCO + RS: TCO vaccinated group challenged with recombinant strain, negative group

The lesion scores were determined and compared between the RS and CS isolates to assess the pathogenicity of the virulent strain detected in Egypt. The different examined tissue sections showed a minimal change at one day post-infection. However, three days post-infection with CEO-like strain (CS) and Recombinant strain (RS) showed an increase in the degree of inflammatory reactions. In the CEO-like isolate infected group, the mean tracheal lesion score was 3.6. The lesions manifested by hyperplasia of glandular goblet cells with inflammatory cells infiltration in submucosa accompanied by vascular reaction as well as catarrhal exudate in tracheal and laryngeal lumen admixed with desquamated epithelium (Fig. 1A). Meanwhile, the recombinant isolate infected group recorded the highest mean lesion score of 4.3 obtained by syncytial cell formation with inflammatory cells hanged in fibrinous exudate in the tracheal lumen (Fig. 2A).

Both CS and RS strains showed an increase in severity at 7 day-post-infection (dpi) detected by the rise in the mean lesion score (Fig. 2B). In the tracheal section of CS infected groups suffered from severe destruction and slouching of the lining epithelium infiltrating mononuclear cells in mucosa with a mean of 4.5. In RS infected group, hemorrhagic patches in the tracheal lumen with severe ulceration of tracheal mucosa were noticed with 5.3 as a mean lesion score (Fig. 1B). In conclusion, it seems that the recombinant isolates infected group showed high pathogenicity compared with CEO-like isolate.

### Histopathological alterations in the trachea of the ILTV vaccinated-infected groups

Histopathological lesion scores were utilized to assess vaccine efficacy against the virulent strains after vaccination. Three days post-infection, the group vaccinated with vector vaccine groups and challenged with CEO-like strain (VEC + CS) showed the lowest mean lesion score of 1.9. Lesions of this group ranged from hyperactivity of goblet cells to inflammatory cells infiltration in tracheal submucosa. While the vector vaccinated group and challenged with RS (VEC + RS) mean lesion score was 2.7. In this group, the tracheal mucosa was thickened by inflammatory cells with hyperactivity of goblet cells and mucous exudate (Figs. 1A and 2A).

However, the CEO vaccinated group and infected with CS (CEO + CS) showed tracheitis with inflammatory cells infiltration, thickened tracheal mucosa, and hyperplastic goblet cells with a mean lesion of 3. Meanwhile, the inflammatory reaction was increased in the CEO vaccinated group who received RS (CEO + RS). This group exhibited catarrhal exudate with deciliation, desquamated epithelium, and inflammatory cells in the tracheal lumen with thickening of its mucosa. The mean lesion score was 3.9. Lesions detected in TCO vaccinated group challenged with CS were similar to the lesions in the CEO vaccinated group and challenged with CS. The mean lesion score was 3. However, the challenged group with RS revealed more severe lesions with an inflammatory reaction, deciliation, and desquamated epithelium. The mean lesion score of 3.6 (Figs. 1A and 2A).

Seven days post-challenge, the tracheal and laryngeal tissues of vector-vaccinated groups infected with CS showed thickening of the mucosa by infiltrating mononuclear cells with hyperplasia of goblet cells and few destructions of lining epithelium with a mean lesion score of 3.2 (Fig. 2B). The group that received RS scored 4.3 as a mean lesion score that detected more deciliation and desquamation of its tracheal and laryngeal mucosa accompanied by exudate and inflammatory cells. Thickening of tracheal mucosa was recorded in vector-vaccinated groups infected with CS more than others challenged with RS. At the same time, deciliation, desquamation, and exudative changes were more in the group challenged with RS (Fig. 1B).

Birds vaccinated with the CEO vaccine and challenged with CS recorded a lower mean tracheal lesion score (3.5) than birds challenged with RS (4). Tracheal tissue sections of the CEO vaccinated CS challenged group revealed mild to moderate catarrhal exudate mixed with inflammatory cells in the tracheal lumen. However, the lesion was more exudative and destructive in the RS group. In the TCO vaccinated group, the mean lesion score of the CS infected group was 3.6, while 5 in the RS infected group. CEO Like vaccine group lesion of CS was exudative and increased in its inflammatory reaction detected by the vascular and cellular response. While the RS-infected group showed destruction and ulceration of the tracheal lining epithelium accompanied by inflammatory cells infiltration and hemorrhage (Fig. 1B).

### Histopathological findings in the harderian gland of the ILTV infected groups

**Fig. 3:**
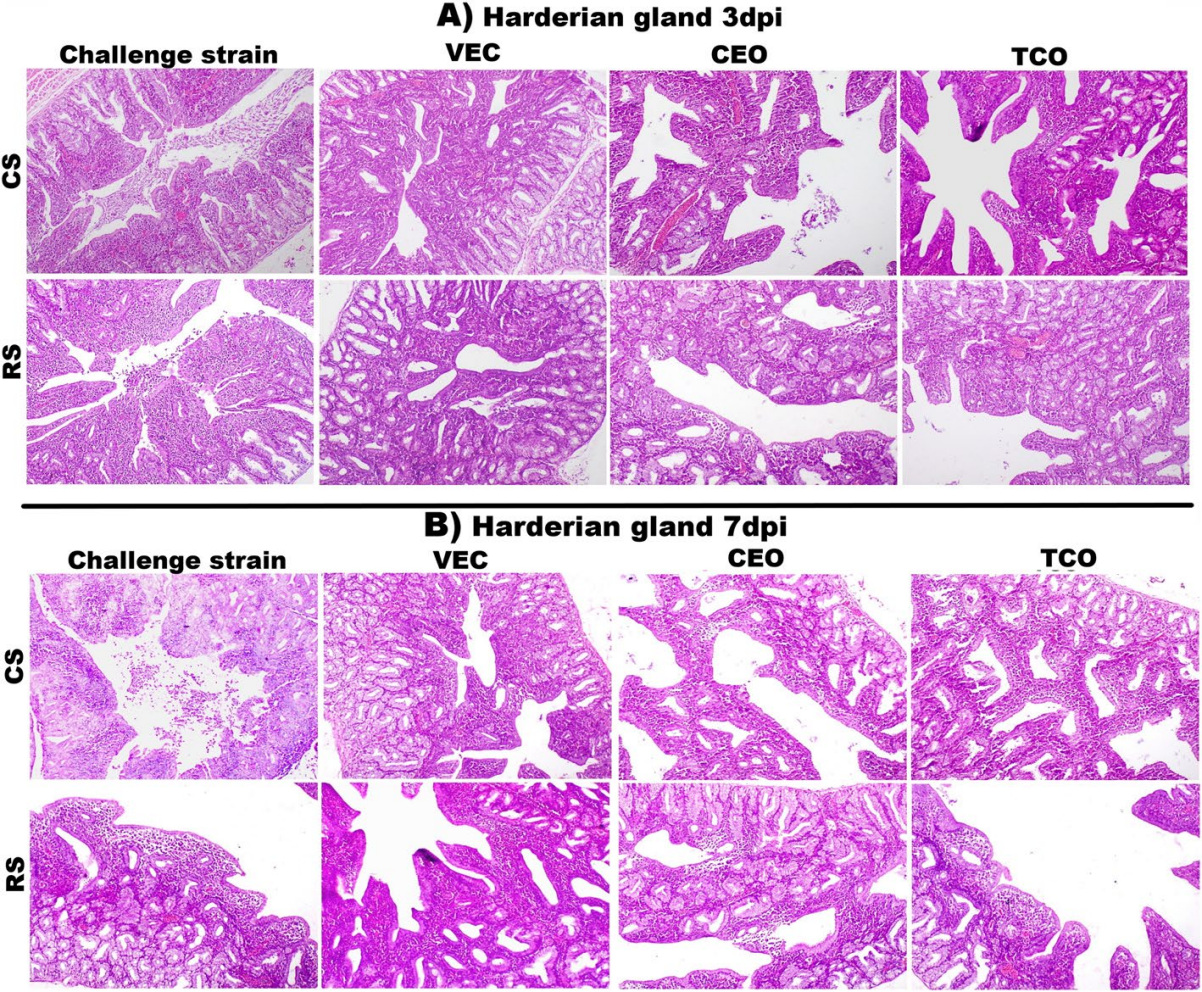
Photomicrographs of H&E-stained Harderian gland sections of the experimental groups: **A** at 3dpi, CS group showing congestion with cellular and exudative cast in harderian gland lumen (H&E,100 X). RS group showing desquamation and necrosis of lining epithelium (H&E,100 X). VEC + CS, VEC + RS, CEO + CS, TCO + CS, and TCO + RS showing mild lymphoid depletion with congestion as well, while CEO + RS showing moderate lymphoid depletion (H&E,100 X). **B** at 7 dpi, CS group showing severe desquamation and necrosis of lining epithelium (H&E,100 X). RS group showing excessive lymphoid depletion with lymphocytolysis (H&E,200 X). VEC + CS showing mild lymphoid depletion (H&E,100 X), VEC + RS showing moderate lymphoid depletion (H&E,100 X); CEO + CS, TCO + CS groups showing moderate to severe lymphoid depletion (H&E,100 X), however CEO + RS and TCO + RS groups showing excessive lymphoid depletion (H&E,100 X). CS: CEO like strain, RS: Recombinant strain, VEC + CS: Vector vaccinated group challenged with CEO like strain, VEC + RS: Vector vaccinated group challenged with recombinant strain, CEO + CS: CEO vaccinated group challenged with CEO like strain, CEO + RS: CEO vaccinated group challenged with recombinant strain, TCO + CS: TCO vaccinated group challenged with CEO like strain, TCO + RS: TCO vaccinated group challenged with recombinant strain

**Fig. 4:**
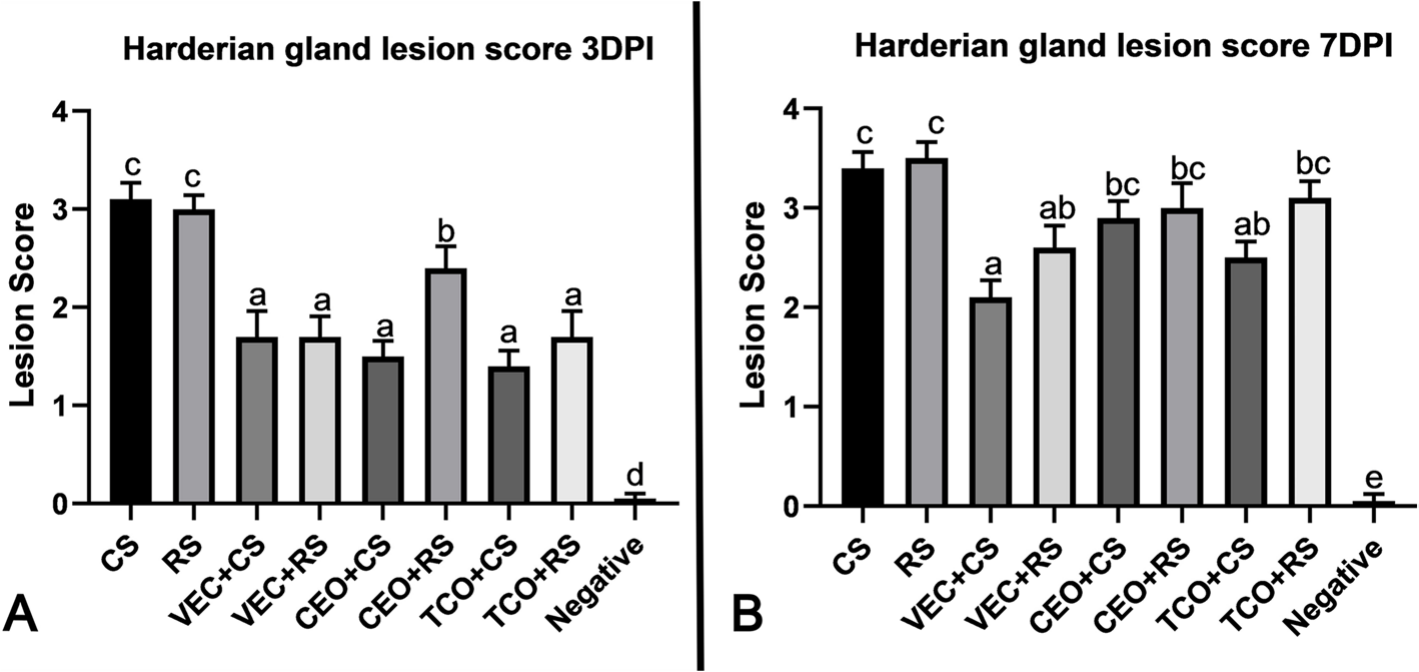
Chart showing Harderian gland score of the experimental groups. **A** at 3 dpi, **B** at 7dpi, Values expressed as means ± SE (error bars), Statistical analyses were performed using a one-way factorial analysis of variance (ANOVA).Means followed by different letters differ significantly when (*P* ≤ 0.05).CS: CEO like strain, RS: Recombinant strain, VEC + CS: Vector vaccinated group challenged with CEO like strain, VEC + RS: Vector vaccinated group challenged with recombinant strain, CEO + CS: CEO vaccinated group challenged with CEO like strain, CEO + RS: CEO vaccinated group challenged with recombinant strain, TCO + CS: TCO vaccinated group challenged with CEO like strain, TCO + RS: TCO vaccinated group challenged with recombinant strain, negative group

Having previously demonstrated that the harderian gland rapidly responds to ILTV infection. We evaluated the pathogenicity and vaccine efficacy in the harderian glands as well. On day one after infection, minimal histopathological alterations of harderian gland were also detected in all examined groups. However, at three dpi, the degree of lesion score was increased in both the CS and RS infected groups. In CS-infected chickens, the harderian gland showed congestion with infiltration of mononuclear cells. The lumen of glands filed with desquamated epithelium and exudate received a mean lesion score of 3.1. while in RS group showed congestion with necrosis and desquamation of lining epithelium that appeared in the lumen with 3 as a mean lesion score (Fig. 3A). However, no significant difference between the two isolates at three dpi (Fig. 4A).

At seven dpi, an increase in lesion scores was detected in both isolates. The lesions displayed in CS-infected chickens ranged from desquamation with necrosed tissue filling the lumen to lymphoid depletion and lymphocytolysis. The mean lesion score was 3.4. While the lesion shown in RS-infected chickens revealed more lymphoid depletion and lymphocytolysis (Fig. 3B) a 3.5 mean lesion score was recorded in this group. In conclusion, the RS-infected group exhibited enhanced pathogenicity in harderian gland compared with CS-infected chickens.

### Histopathological alterations in the harderian gland of the ILTV vaccinated- infected birds

There was no significant difference between all vaccinated challenged groups at three dpi except the CEO vaccinated group challenged with the recombinant isolate. Vector-vaccinated groups that were challenged with both strains recorded the same mean lesion score (1.7). These groups detected congestion with a mild degree of lymphoid depletion. In TCO vaccinated groups, there was no significant difference between the two challenge strains, CS or RS, with a mean lesion score of 1.4 and 1.7, respectively. Mild lymphoid depletion was detected in TCO + CS that began with mild to moderate depletion in TCO + RS. However, there was a significant difference between both strains as recombinant strain in CEO vaccinated groups showed more lesions that scored 2.5 other than CEO-like strain that scored 1.5 as in CEO + RS group moderate to severe lymphoid depletion were observed in harderian gland (Fig. 3A).

Seven dpi, there were no significant differences between both strains in vector-vaccinated challenged chickens but numerically increased in the recombinant strain as VEC + CS was 2.1 and VEC + RS was 2.6. Mild to moderate lymphoid depletion was observed in both groups. The CEO vaccinated challenged groups showed increased lesion scores in both strains, 2.9 in the CEO + CS group and 3 in the CEO + RS group, as moderate depletion was detected in both groups. However, in TCO vaccinated group, the lesion score increased from 2.5 in TCO + CS to 3.1 in TCO + RS because of increased lymphoid depletion (Figs. 3B and 4B).

### Viral load quantitation

**Fig. 5:**
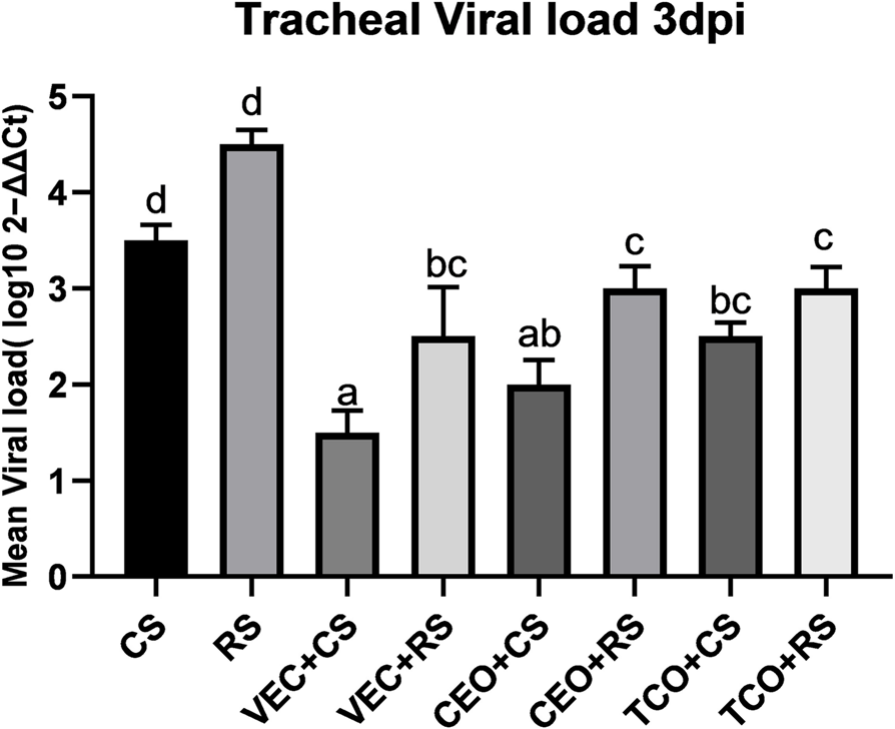
Relative quantitation of viral genome loads in trachea after three dpi. Mean viral genome loads were expressed as the 2 − ΔΔCt standard error of the mean (SEM) post-challenge. Statistical analyses were performed using a one-way factorial analysis of variance (ANOVA). Different letters (a-d) indicate significant differences (*P* ≤ 0.05).CS: CEO like strain, RS: Recombinant strain, VEC + CS: Vector vaccinated group challenged with CEO like strain, VEC + RS: Vector vaccinated group challenged with recombinant strain, CEO + CS: CEO vaccinated group challenged with CEO like strain, CEO + RS: CEO vaccinated group challenged with recombinant strain, TCO + CS: TCO vaccinated group challenged with CEO like strain, TCO + RS: TCO vaccinated group challenged with recombinant strain

The presence of ILTV DNA was evaluated by real-time qPCR in tracheal swabs collected three days post-challenge. As expected, no viral genome loads were detected in swabs collected from non-vaccinated non-challenged chickens. In contrast, 1.5–4.5 fold change genome loads were detected in tracheal swabs samples from challenged chickens (Fig. 5). The mean viral load in CS and RS groups was 3.5 and 4.5, respectively. This fold change was declined in the vaccinated challenged group as in VEC + CS was 1.5, VEC + RS was 2.5 while in CEO vaccinated groups was 2 in CS infected group that elevated to 3 in RS infected chickens. In comparison, the TCO + CS was 2.5 which elevated to 3 in RS challenged group. To sum up, the RS- infected group recorded the most remarkable fold change, and the vector-vaccinated group exhibited better protection than live vaccines.

## Discussion

The objective of the study was to determine the pathogenicity of recently GaHV-1 strains we have detected and characterized from recent outbreaks in Egypt. Additionally, we aim to evaluate the protection level of various commercial vaccines against these strains. The pathogenicity and vaccine efficacy were assessed by histopathological lesion score of tracheal and harderian gland along with the determination of viral shedding through quantification of UL44 (gC) viral load. One day post-infection/challenge, minimal changes were observed in tracheal and harderian gland tissues with no significant difference between all test groups. Whereas three dpi significant differences were detected between CS and RS. The RS group showed severe tracheal lesions with a mean of 4.3 while tracheal viral load was 4.5-fold change. The CS group mean lesion was 3.6 and viral load was 3.5 fold change. These findings agreed with the previous records stating that potential recombinant strains are much more aggressive reactions in SPF chickens [21, 25, 26]. However, the virulence factors responsible for this aggressiveness were not determined in this study and warrant further investigation in the near future.

In vaccinated groups, the lowest tracheal lesion and viral load were detected in VEC + CS. This notion could be explained through several studies that recorded the lack of horizontal transmission with improved performance and diminished clinical signs in birds administered with FPV-LT and HVT-LT vaccines compared with CEO and TCO live vaccines [16–18]. In contrast, the VEC + RS group exhibited increased tracheal lesion and viral load. This result could also be explained due to increased virulence of the recombinant strain as previously detected in various outbreaks [21]. These results augment the notion that the recombinant strain can cause immune evasion. However, this study did not discuss the mechanism of this immune evasion. Additionally, CEO and TCO vaccinated groups showed no statistical difference against CEO-like strain. While lesions increased in CEO and TCO vaccinated groups received RS compared to CS, which was confirmed by tracheal viral genome load.

In seven-day post-infection, tracheal lesions were increased in severity in both strains. In recombinant-infected groups, the mean was numerically increased from (4.3/0.15) at 3dpi to (5.3/0.15) at 7dpi. Similarly, in CEO like-infected group, the mean was elevated from (3.6±0.16) at 3dpi to (4.3±0.15) at 7dpi. As reported previously, virulence and viral load are elevated with time progression [24]. Furthermore, the range of clinical signs and lesions that vary in severity depends on the infecting strain [21, 27]. Agnew-Crumpton et al., [25] reported that recombinant viruses are more virulent than previously dominant ILTV strains.

However, vaccinated groups showed no significant difference in the vector and CEO vaccine that challenged both CS and RS. At the same time, TCO-vaccinated groups infected with CS and RS showed the most severe lesion score among vaccinated groups related to the TCO vaccine is much less immunogenic than vector and CEO vaccines as previously investigated Palomino-Tapia et al., [28]. The latter study showed that the TCO vaccine reduced clinical signs and virus replication but conferred only partial protection with time progression when compared with vector and CEO vaccines.

Although harderian gland is not connected anatomically to the respiratory system, it harbors well organized lymphoid follicles and intraepithelial lymphocytes. Therefore, harderian glands act as innate barriers to antigens [29]**.** It has been suggested that macrophages may assist in spreading the virus to other non-respiratory sites [27]. Moreover, it was reported that harderian gland was organized to rapidly respond to ILTV infection by the early and transient upregulation of the IFN-γ gene one day post-infection as investigated else were to validate the examination of harderian gland to evaluate the virulence of ILTV isolates [24]**.** Furthermore, the harderian gland is considered a primary source for ILTV uptake alongside the nasal cavity and conjunctiva, which possibly reflect a pivotal role in early replicates and viral fate [30]. Additionally, others confirmed that the harderian glands had a sustained increase in inflammatory cells [31].

Harderian gland at three dpi showed no significant difference between CS and RS lesion as congestion, infiltration of mononuclear cells, and desquamation were observed in both groups. It could be explained that the harderian gland of ILTV-infected chickens had a sustained increase of inflammatory cells related to early and transient gene transcription of IFN-γ [24]. All vaccinated groups showed no significant difference between both challenge strains. Mild to moderate lymphoid depletion was detected, except in the CEO + RS group, which increased lymphoid depletion.

Increased lesion scores were detected in both strains at seven dpi. Their mean lesion score was 3.4. While in RS infected group was 3.5 in which harderian gland showed more lymphoid depletion and lymphocytolysis as recorded as well in Krunkosky et al., [31]. The latest group reported that the most notable cellular change was the decrease of plasma cell numbers on 7 and 9 dpi with ILTV. Both challenged groups observed mild to moderate lymphoid depletion in vector-vaccinated chickens. However, CEO vaccinated groups showed increased lesion scores with moderate depletion in both groups. In TCO vaccinated group, the TCO + RS group showed an increase in lymphoid depletion, unlike the TCO + CS group.

It is possible that recombination may have been facilitated by the conditions under which the ILT vaccines were used, including the mass delivery of multiple vaccines to large numbers of intensively housed birds. This recent finding highlights the risk associated with the use of numerous attenuated ILT vaccines under conditions imposing high selective pressures, which may foster recombination between co-circulating viruses and selection of more virulent or transmissible progeny [14, 32]. This is the first study conducted to evaluate commercial ILTV vaccines against recently characterized ILT viruses in Egypt. However, further work is needed to support these findings by testing other commercial vaccines locally and worldwide. Ultimately, it is also essential to improve current vaccine administration protocols and develop new protocols to enhance flock immunity and disease control.

## Supplementary Information

**Additional file 1: Supplementary Figure.** Microphotograph is showing normal chicken embryo fibroblast (CEF) in the negative control while the positive ILTV infected CEF is showing cytopathic effect of the virus manifested by clumps and aggregates of cells (circle).

## Data Availability

All data used during the current study are available in the article.
