# Supplementary material for: Pathogenicity and vaccine efficacy of two virulent infectious laryngotracheitis virus strains in Egypt
Source: BMC Vet Res. 2022 Sep 26;18:358. doi: 10.1186/s12917-022-03458-3 (PMC9511729; doi:10.1186/s12917-022-03458-3)

**Suplementary Figure : Microphotograph is showing normal chicken embryo fibroblast (CEF) in the negative control while the positive ILTV infected CEF is showing cytopathic effect of the virus manifested by clumps and aggregates of cells (circle)**


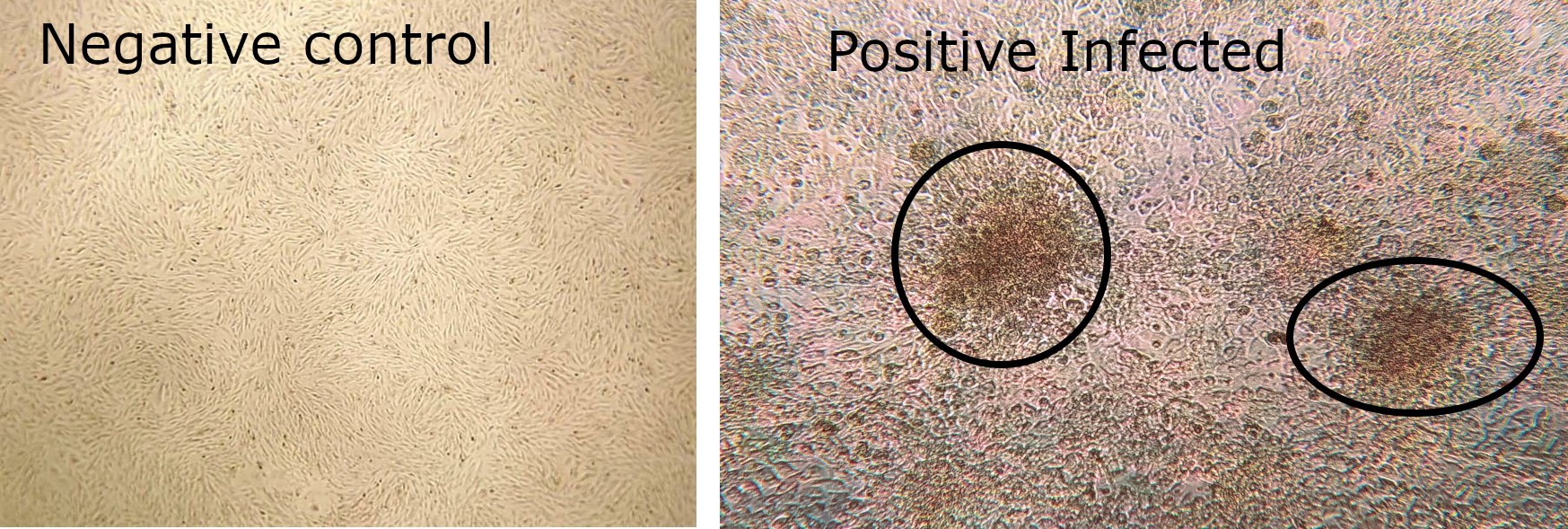

Supplement: Supplementary file 1 — Additional file 1: Supplementary Figure. Microphotograph is showing normal chicken embryo fibroblast (CEF) in the negative control while the positive ILTV infected CEF is showing cytopathic effect of the virus manifested by clumps and aggregates of cells (circle). [file 12917_2022_3458_MOESM1_ESM.docx]
